# Army Dentists

**Published:** 1899-06

**Authors:** Morris I. Schamberg


### ARMY DENTISTS.

BY MORRIS I. SCHAMBERG, D.D.S., M.D.[1]

The late war has brought to light many improvements, ay, necessities, that are requisite to a successful military campaign. Furthermore, it has proved that it is not best to await the outbreak of actual warfare before adding such necessities. Our regular army was found to be too small, various departments were proportionately so, and there was a total absence of departments that would have added materially to bring our army nearer the state of perfection. The fact that there was no department of dentistry, and that there was great need for that addition, was forcibly impressed upon the mind of Representative Hull, with the resultant clause to his bill, calling for the appointment of one hundred dentists. This bill not having been favorably acted upon as yet, allows for a free discussion of the advisability of starting this particular branch of the service.

Numerous articles have been written for and against the establishment of this department, for it must be remembered that the subject was receiving considerable attention even prior to the late agitation of military affairs.

It was, indeed, surprising to read in a recent edition of a Western dental journal, the opinion on this matter of a man hold-

---

[1] Late acting assistant surgeon, United States army.

ing the degree of doctor of dental surgery, and who had served as a major in the volunteer service. The writer has proceeded to set up ridiculous reasons for the proposed legislation, and then, in demolishing them, has thought that he has controverted all arguments in its favor.

The writer says, "The consideration that appeals most strongly to the profession is the opportunity given to recent graduates and young dentists with limited practice to secure remunerative employment."

He further states that "the departments of the government for war are not organized for charity or to give employment to those otherwise unable to succeed in a profession of their own choosing." These allusions that he makes to the profession trying to force its services upon the government, for the purpose of giving profitable occupation to dentists, are too sordid to deserve any notice whatsoever. While a few men might have written encouragingly about the advisability of organizing a department of dentistry for the army and navy, and in such articles had spoken of the benefits the young practitioner would derive from the adoption of such a department, no one with a reasonable amount of judgment would suppose for one moment that the government would be induced to go to such a great expense without seeing the necessity for such a measure.

Is it not natural for the profession, knowing the necessity for the proper care of the teeth and mouth, knowing the relation between the condition of the masticating organs and the general health, knowing that every soldier at some time or other requires advice or treatment for these parts,—is it not natural that it should be disposed to encourage the adoption of the army and navy dental surgeon? It is the profession's duty to depict its possibilities in that direction, to show its necessity, and to suggest the proper procedure for the establishment of an army dental department.

While serving at Chickamauga, many soldiers came to me for the extraction of teeth, and many more could have been relieved had I had proper filling-materials and instruments with me. Nothing tends to bring any condition in the mouth to a crisis as does the life that a soldier leads in the field. Exposed to all kinds of weather, run down through fatigue, dependent upon rations which, as a rule, put to a severe test the masticating organs, and non-painstaking as to the hygiene of his mouth by reason of this peculiar life, the soldier proves to be the most needy consultant of the dentist.

During six and one-half months of active service in Porto Rico, many cases of interest from a dental stand-point presented themselves to me. Rapidly growing alveolar abscesses were of frequent occurrence, and one case of rather extensive necrosis of the inferior maxilla called for operation. The above-mentioned cases, together with numerous conditions of the mucous membrane which called for treatment, such as ulcerative stomatitis, spongy gums, chancre of the tongue, etc., are proof of the fact that the vicissitudes to which the soldier is subjected render him an easy victim to some of the more serious oral affections.

The question is often asked, Cannot the physician do the necessary extraction of teeth? You might just as well ask, Cannot a dentist treat and confine a pregnant woman? Both have about an equal knowledge of the duty they are called upon to perform, and in both instances does the patient suffer the uncertainty of success.

Again the query is put, Why should not the soldier be referred, as in the past, to the dentist in the neighboring town or city; and again we might add, for the same reason that he is not thrown upon his own resources and sent to a physician of the adjacent districts when medical attention is needed. But the opposition says, the medical man is needed in time of war and the dental man is not, for he would be unable on account of the manœuvres of war to do other than extracting. In answer, I will take the liberty of stating that the dentist in times of peace would be much busier than the physician in preparing the soldier for the test of war. There is scarcely an individual that at some time does not require the services of a dentist. One can then realize how much work would devolve upon a dentist in care of a regiment of a thousand men.

Not alone the scientific thinker, but the most practical practitioner, attributes a large proportion of gastric and intestinal disorders to defective mastication of food. During the late war, this was found to be the most troublesome class of diseases.

Our Western friend assumes that the standard of the dental man will necessarily be a poor one. The stringent examinations given in all the departments of the government service will preclude the possibility of any improperly trained dentists getting into the service. Should a man prove to be unworthy of the position, he must expect to be treated as in other walks of life, with little consideration. A conscientious man of gentlemanly and dignified instincts entering the army with but "forceps rampant for a collar ornament" will carry with him all the respect that any man of character will in other departments, and I predict that if he be a man of ability, as he necessarily should be, the officers and their families will supply a *not* very small part of his practice.

True, the army dentist will share with other members of the service some leisure moments. These can be advantageously spent in acquainting himself with principles of surgery, and in consequence of this advanced knowledge the army dentist will be able to lend much assistance upon the battle-field. There are many other duties which he might be called upon to perform in common with the surgeon, such as the instruction of the hospital corps in drill regulations and points of discipline.

As the medical men are encouraged to perform original research work and thereby advance the standing of their profession, so would the dental men have ample opportunity to collect and report interesting statistics as to the wearing qualities of certain filling-materials, the advantage of one alloy or cement over others, etc. Their cases would be constantly before them, and the uniform employment throughout the service of certain preparations would afford most valuable information as to their efficiency. So, while a man may not be tied down to actual office practice the entire day, he will have many other duties to perform which will keep him busy.

There are a few points about the establishment of an army dental department that suggest themselves to me. One is that the department should, without doubt, be in close relation with, if not directly under, the jurisdiction of the medical department. Dentists should be taken into the service with the rank of second lieutenant, giving an opportunity to the most worthy to rise by promotion to the position of captain or even major. Dentists who hold the degree of medicine should be given the preference of appointment, in that they can be called upon in an emergency to perform the duties of a surgeon.

